# Idiopathic CD4 T Cell Lymphocytopenia: A Case of Overexpression of PD-1/PDL-1 and CTLA-4

**DOI:** 10.3390/idr13010009

**Published:** 2021-01-13

**Authors:** Gaurav Kumar, Heidy Schmid-Antomarchi, Annie Schmid-Alliana, Michel Ticchioni, Pierre-Marie Roger

**Affiliations:** 1 Arthritis and Clinical Immunology, Oklahoma Medical Research Foundation, 825 NE 13th Street, Oklahoma City, OK 73104, USA; 2 Unité 576, Institut National de la Santé et de la Recherche Médicale, Hôpital de l’Archet I, 151 Route de Saint-Antoine, 06200 Nice, France; heidy.SCHMID-ANTOMARCHI@unice.fr (H.S.-A.); Annie.SCHMID-Alliana@unice.fr (A.S.-A.); ticchioni.m@chu-nice.fr (M.T.); pierre-marie.roger@chu-guadeloupe.fr (P.-M.R.); 3 Laboratoire d’Immunologie, Hôpital de l’Archet, Centre Hospitalier Universitaire de Nice, 151 Route de Saint-Antoine, 06200 Nice, France; 4 Infectiologie, Centre Hospitalier Universitaire de la Guadeloupe, Pointe-à-Pitre/Abymes, Route de Chauvel, Les Abymes, 97139 Guadeloupe, France; 5 Faculté de Médecine, Université des Antilles, 97157 Pointe-à-Pitre, France

**Keywords:** Idiopathic CD4 T cell lymphocytopenia, T cell, co-stimulatory molecules, PD-1, PDL-1, CTLA-4

## Abstract

Idiopathic CD4 T cell lymphocytopenia (ICL) is a rare entity characterized by CD4 T cell count of <300 cells/mm^3^ along with opportunistic infection for which T cell marker expression remains to be fully explored. We report an ICL case for which T lymphocyte phenotype and its costimulatory molecules expression was analyzed both ex vivo and after overnight stimulation through CD3/CD28. The ICL patient was compared to five healthy controls. We observed higher expression of inhibitory molecules PD-1/PDL-1 and CTLA-4 on CD4 T cells and increased regulatory T cells in ICL, along with high activation and low proliferation of CD4 T cells. The alteration in the expression of both the costimulatory pathway and the apoptotic pathway might participate to down-regulate both CD4 T cell functions and numbers observed in ICL.

## 1. Introduction

Idiopathic CD4 Lymphocytopenia (ICL) is a syndrome first defined in 1992 by the Centre for Disease Control and Prevention (CDC) of the USA as a documented absolute CD4 T lymphocyte count of less than 300 cells per cubic millimeter, or of less than 20% of total T cells on more than one occasion, usually two to three months apart, no evidence of infection on HIV testing, and the absence of any defined immunodeficiency or therapy associated with depressed levels of CD4 T cells [1,2,3]. The clinical presentation is characterized by a variety of opportunistic infections and autoimmune manifestations [3,4,5,6,7].

Heterogeneous immunologic profiles have been reported so far in ICL patients. Therefore, no single theory for the pathophysiology of ICL has been formalized yet. There seems to be a remarkable change in the CD4 T cells count, whereas CD8 T cells remain unchanged or often decreased in ICL along with defective cell mediated and humoral immunity [3,8,9]. Decreased T cell responses to T cell receptor stimulation (TCR) as well as increased T cell activation have been reported in earlier works [5,10,11]. Moreover, in some ICL patients, a defective T cell proliferation ability is seen specifically for CD4 and not for CD8 T cells [12,13]. Enhanced apoptotic depletion of CD4 T cells is also considered to be the cause for low pool of circulating T cells [12]. Accordingly, our team had earlier reported an overexpression of Fas/CD95 and Fas-induced apoptosis in ICL patients. This significant observation was seen for CD4 T cells only [14]. Zonios et al. reported a marked alteration in activation and turnover markers only on CD4 T cells, indicating a defective CD4 T cells compartmentalization in ICL patients [5]. Reports of increased regulator T cells (T-regs), may also explain, at least in part, T cell anergy and ultimately enhanced apoptosis level seen in ICL [12].

Till date, the expression of costimulatory molecule in idiopathic CD4 T cell lymphocytopenia has not been reported in spite of to their role in antigen-driven proliferation and/or in lymphopenic conditions [15,16]. Mechanistic studies suggest that CD3-TCR engagement leads to activation and proliferation of T cells through a series of biochemical events [17]. Indeed, CD28 promotes T cell proliferation, whereas CTLA-4 engagement results in T cell unresponsiveness to stimulatory signals.

Herein, we have reported a new case of ICL, characterized by highly activated CD4 T cells, and increased T-regs along with altered CTLA-4 and PD-1/PDL-1 expression, which globally converge for CD4 T cell unresponsiveness and lymphocytopenia.

## 2. Materials and Methods

### 2.1. Patient

The patient was a 60 years-old female who was hospitalized in our department for severe sepsis related to *Staphylococcus aureus* bacteremia. The port of entry was unknown but the patient presented with secondary arthritis of the right knee, a muscular abscess of her right *musculus soleus*, and a severe skin and soft tissue infection of both legs. She did not have endocarditis and her severe sepsis was resolved after surgery and antibiotic therapy. Due to the septic arthritis and surgical drainage of the muscular abscess, the patient benefited of a prolonged clinical and biological follow-up during which severe lymphopenia was constantly observed < 300/µL, with a normal CD4/CD8 ratio despite normal leucocytosis. This depleted CD4 T cell count persisted for 60 months. The serum levels of IgG, IgM, and IgA were within the normal range. It should be noted that the patient suffered a multimeric dermatomal varicella zoster virus infection three years later. As virological and immunological investigations failed to demonstrate a given etiology to this severe lymphopenia, we concluded that the patient suffered an idiopathic T cell lymphocytopenia after which we explored her immunological status further upon her consent.

### 2.2. Sample Processing and Cell Culture

Blood samples was drawn from the patient in tubes containing acid citrate dextrose (ACD) from Infectious Disease Department of the Nice University Hospitals, France and brought to lab within an hour to perform the experiments. Five healthy controls (HC) were also included, whose recent routine clinical checkup reports were normal. Informed consent was obtained from the patient and all the healthy donors and the study was conducted in accordance with the ethical committee of our institute.

Peripheral blood mononuclear cells (PBMCs) were isolated from the blood using Ficoll-paque PLUS (STEMCELL Technologies, Grenoble, France) density gradient centrifugation. For this, 10 mL of whole blood was diluted to 20 mL with RPMI 1640 (Thermo-Fisher Scientific (Gibco), Illkirch-Graffenstaden, France) and layered on 10 mL of Ficoll-paque PLUS slowly without mixing the two in a 50 mL falcon, which was then centrifuged at 400× *g* for 30 min. PBMCs were separated as a ring layer at the interface, which was carefully removed and washed. For analysis of phenotypic and functional markers, PBMCs were used immediately after their isolation and after overnight stimulation with CD3 and CD28 at 37 °C with 5% CO_2_ in RPMI 1640 supplemented with 10% of fetal calf serum (FCS).

### 2.3. Cell Stimulation with CD3/CD28

Cell culture was performed in wells coated with anti-CD3, and anti-CD28 mAbs added in solution (clone 28-2, 10 μg/mL final dilution). Wells were coated as follows: 500 µL of medium containing CD3 mAbs (clone X3, 10 µg/mL final dilution), incubated for 2 h at 37 °C and washed 3 times with the medium alone. Clone X3 and 28-2 were produced in our laboratory.

### 2.4. Antibodies

The following conjugated Anti-human monoclonal antibodies (mAbs) were purchased from BD Pharmingen (San Jose, CA, USA): CD3 FITC (clone SK7), CD3 PE-Cy7 (clone SK7), CD4 FITC (clone SK3), CD4 PerCP-Cy5.5 (clone SK3), CD4 APC (clone SK3), CD8 PerCP-Cy5.5 (clone SK1), CD25 PE (clone 2A3), CD45RA FITC (clone L48), CD62L APC (clone SK11), CD80 FITC (clone BB1), CD86 PE (clone 2331), CTLA-4/CD152 PE (clone BNI3), HLA-DR APC (clone L243), HLA-DR PE-Cy7 (clone L243), Ki67 PE (clone B56), FITC Annexin V Apoptosis Detection Kit, Fas PE (clone DX2), FasL Biotin (clone NOK-1), Streptavidin PE-Cy7, and Propidium Iodide. CD28 FITC (clone CD28.2), CD45 Pacific blue (clone HI30), PD-1 PE (clone MIH4), PDL-1 APC (clone MIH1), and FoxP3 APC (clone 236A/E7) were purchased from eBioscience, Inc., San Diego, CA, USA.

### 2.5. Multiparameter Flow Cytometry

For extracellular labeling of the cells, one million PBMCs were taken in tube and antibodies were added to it. Thereafter, cells were incubated at 4 ℃ in the dark for 30 min, washed twice with RPMI, and analyzed using flow cytometry (FACS CANTO II, Bec, Becton Dickinson, Franklin Lakes, NJ, USA) within 24 h of labelling. For intracellular labelling, the cells were firstly labelled with extracellular antibodies, washed in RPMI, fixed and permeabilized using intracellular FACS kit (eBioscience). Cells were then stained with mAbs with an isotype-matched control mAbs (Becton Dickinson) for each marker using the same flurochrome. These nonspecific control mAbs were used to position the quadrants and correctly gate out the positive population. A minimum of 2500 CD3+ cells (T cells) were recorded each time for analysis of markers on the cell population. We used cytofluorometric techniques with a six-colors labeling pattern (fluorescein isothiocyanate (FITC)/phycoerythein (PE)/peridinin chlorophyll (PerCP) cyanin (Cy) 5·5/PE-Cy7/allophycocyanin (APC)/Pacific blue) and the data was analyzed using FlowJo software (Tree Star Inc., integrated into FACSCanto II and supplied by Becton Dickinson)

## 3. Results

**Figure 1:**
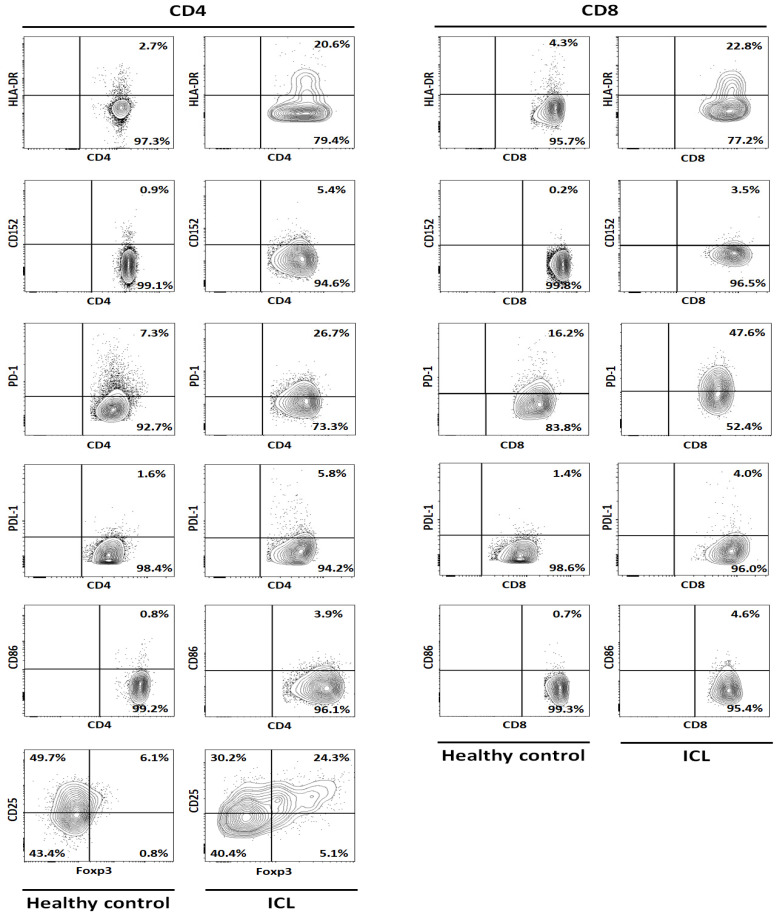

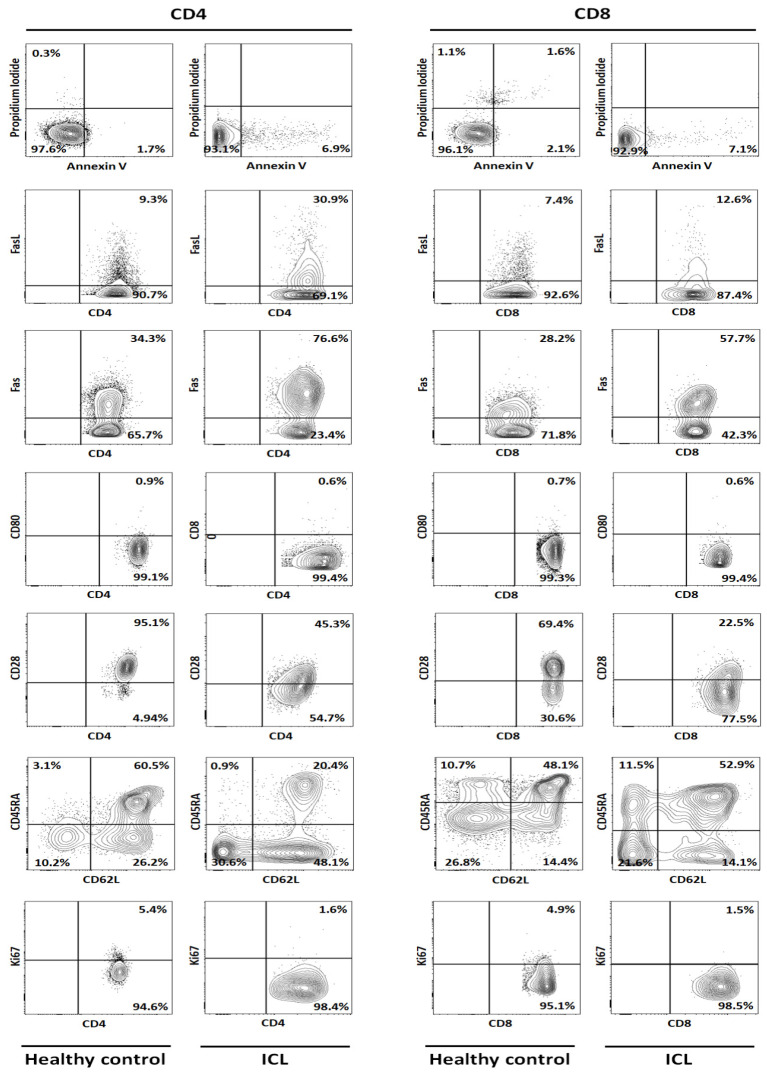
T cell phenotype from idiopathic CD4 T cell lymphocytopenia (ICL) patient and healthy control. Representative flow cytometric data of markers for activation (HLA-DR+), costimulatory and inhibitory molecules (CD28, CD80, CD86, CD152, PD-1, PDL-1), regulatory T cells (CD25+Foxp3+), apoptosis (AnnexinV+Propidium iodide, Fas, FasL), naïve T cells (CD54RA+CD62L+), and proliferation (Ki67+) expressed on CD4 and CD8 T cells from ICL patient and healthy control. First, the living population was gated on total cells analyzed. Then, on living CD45+ cells, CD3+ cells were gated and finally CD4 and CD8 T cells were gated on CD3+ cells.

We compared the ICL patient to 5 HC (mean age ± std dev: 49 ± 19 years) chosen from our voluntary staff in order to characterize the immunological disorder of the patient. Due to the limitation of statistical analysis between one ICL patient and five controls, we arbitrarily defined the values to be significant when there was at least a threefold difference between the two. The T cell phenotype we analyzed is described in Table 1 and Figure 1, the markers being chosen in accordance with previous reports on ICL and in other human diseases [7,10,11,12,14,18,19,20,21].

We observed a significant upregulation of the activation marker HLA-DR on both CD4 and CD8 T cell subset, with CD4 being almost 2-fold higher than CD8 T cells, in the ICL patient as compared to healthy controls. Moreover, there was almost a 4-fold increase in the expression of PD-1 and its ligand, PDL-1 on CD4 T cells in the patient in comparison to controls. This significant increase of PD-1 and PDL-1 was not observed for CD8 T cells.

For regulatory T cells, as measured by the expression of CD25 and Foxp3 on CD4 T cells, we observed an increase of 316% in ICL in comparison to HC. This was associated with an increase in apoptosis, measured using Annexin V marker, by 311% of CD4 T cells and 234% for CD8 T cells in the patient in comparison to HC. We did not observe any differences in the expression of Fas and its ligand FasL on neither CD4 nor CD8 T cells in this ICL patient as compared to the controls.

As increased T cell activation and senescence could be an indicative of an altered T cell receptor signaling, we looked for the expression of costimulatory molecules in our ICL patient. We observed a 5-fold increase of CD152/CTLA-4 on CD4 T cells and 2.5-fold increase on CD8 T cells in ICL patient as compared to HC. This was associated with almost 3-fold increase of CD86 on both CD4 and CD8 T cells in ICL patient as compared to controls. The expression of other costimulatory molecule, mainly CD28 and CD80 was not significantly altered on either CD4 T cells or CD8 T cells in ICL patient in comparison to controls.

To determine whether the low number of CD4 T cells in our patient could be associated with a defective proliferation ability, we looked for the expression of Ki67, a marker for proliferation in T cells. We found a significantly reduced CD4 T cell proliferation with a 4-fold decrease for CD4 T cells and 2.6-fold by CD8 T cells in the ICL patient as compared to controls. T cell proliferation is often associated with T cell expansion and a decreased T cell proliferation in our ICL patient suggested towards a decrease in the naïve T cell pool. Therefore, we also looked for the naïve T cell population represented by the double expression of CD45RA and CD62L. We observed almost 3-fold decrease of naïve CD4 T cells, in the ICL patient as compared to HC. This decrease of naïve T cells was not observed for CD8 T cells. This trend towards a deactivation phenotype of CD4 T cells was not significantly altered after overnight stimulation (data not shown).

## 4. Discussion

Our present report on an ICL patient is the first showing an overexpression of PD-1 and PDL-1 along with increased CTLA-4 expression, on CD4 T cells. In addition, increased T-regs found in the ICL patient was an indicative of a suppressed T cell repertoire. These altered apoptotic and costimulatory pathways together contribute to the suppressed and low pool of circulating CD4 T cells in the ICL patient.

Our study has some limitations. Firstly, due to the limited availability of the sample, we were not able to study other important markers for T cells, such as chemokines, cytokines, etc. Secondly, we were able to only do the phenotypic characterization of T cells and, therefore, the cellular and molecular kinetics behind low CD4 T cell counts in our ICL patient could not be very well explained.

ICL is considered to be a multifactorial disease and, therefore, a great heterogeneity in the clinical presentation as well as T cell abnormalities has been reported so far. Previous reports on ICL patients suggest a failure of regenerative capacity of hematopoietic stem cells in the bone marrow as the cause of CD4 T cell depletion [10]. However, Hubert et al. and others demonstrated that a defective T cell receptor signaling and renewal capacity of peripheral CD4 T lymphocytes might participate in CD4 T cell lymphocytopenia [12,13]. Accordingly, we looked for the abnormalities in peripheral T cells of our new ICL patient, considering them to be the cause of low pool of CD4 T cells in the circulation.

ICL is usually associated with T cell activation and enhanced apoptosis, particularly for CD4 T cells [5,14]. In a prospective study, Zonios et al. have reported a significantly higher proportion of activated (HLA-DR+) CD4 T cells in ICL patients, but not of CD8 T cells [5]. In this study, we also report a similar trend of an activated CD4 T cell profile using the same activation marker along with increased T cell apoptosis in an ICL patient. In addition, previous studies also reported a higher percentage of circulating T-regs in ICL, which we also observed in our patient [5]. Accordingly, our new case of ICL fit these characteristics.

Activation induces the expression of inhibitory receptors, CTLA-4, to inhibit proximal T cell receptor and other downstream signaling to regulate self-tolerance [22]. Therefore, we also looked for abnormalities in the inhibitory and costimulatory molecules in our ICL patient. We observed an alteration of the costimulatory molecule, an overexpression of CTLA-4 only on CD4 T cells and CD86 on both T cell subsets, while CD28 and CD80 were similarly expressed in the ICL patient as compared to controls. CTLA-4 plays an essential role in immunologic homeostasis as a negative regulator of T cell activation. However, little is known about the regulatory mechanism for CTLA-4 expression. T cell activation is generally self-limited, in part because activated T cells express receptors, such as CTLA-4 that mediate inhibitory signals from the antigen presenting cell [23]. CTLA-4 binds CD80 or CD86, the same ligands that signal positively through CD28 [24,25]. It has been reported that in vitro ligation of CTLA-4 during T cell stimulation blocks activation, cytokine release, and proliferation in humans [26]. Thus, overexpression of CTLA-4 may be the result of general activation of T cells but may also play a role, at least in part, in enhanced CD4 T cell apoptosis in the patient.

PD-1 is another important inhibitory molecule, which is overexpressed to prevent hyper-activation of T cells [22]. The pathway consisting of the receptor PD-1 and its ligands PDL-1, is a newer pathway in the B7-CD28 family that regulates the balance between stimulatory and inhibitory signals needed for effective immunity and the maintenance of self-tolerance [27,28]. PD-1 is expressed by apoptotic cells and its overexpression could increase the classical programmed cell death [29]. Moreover, PD-1 signals are inhibitory but appear weak and are more obvious when positive signals are blocked [30]. Moreover, to the best of our knowledge, this is the first report showing increased PD-1 and PDL-1 on CD4 T cells in an ICL patient.

It is now well established that T-regs are indispensable for the maintenance of immunologic self-tolerance and immunosuppression [31]. However, it should be considered that T-regs are also a specialized subpopulation of CD4 T cells that exert a suppressing effect on T cells, including inhibition of proliferation [32]. In an interesting experiment using a mouse model, Yannick et al. reported that immunosuppressive effects of streptozotocin-induced diabetes results in absolute CD4 T cell lymphopenia and a relative increase of T-regs [33]. Thus, in our case, increased T-regs in our ICL patient, +316% compared to controls, could be seen as another cause of low proliferation rate of CD4 T cells.

Inhibitory molecules, PD-1 and CTLA-4, have been shown to act in a complimentary manner in maintaining peripheral T cell response [34]. CTLA-4 predominantly regulates the initial phase of the T cell response whereas PD-1/PDL-1 mainly affects the late phase and functions to maintain the anergic state of T cells [35]. Francisco et al. have already reported that PD-1 and PD-L1 ligation on CD4+ T-regs support T-reg stability and expansion [36]. In vitro studies have also shown that engagement of PD-1 by its ligand inhibits proliferation and guide the differentiation of Th1 cells into T-regs [37,38,39]. Therefore, increased T-regs in our case might be associated with increased PD-1 and PDL-1 expression. [37]. Moreover, CTLA-4 partially regulates the suppressive function carried out by T-regs [40]. Taken together it may be considered that increased PD-1 and PDL-1 expression along with CTLA-4 may directly act to inhibit the proliferation of CD4 T cells or increased T-regs, which in turn also lead to reduced proliferation but increased apoptosis of CD4 T cells, consequently leading to ICL.

In conclusion, it is unlikely that a single pathophysiology will be operative in ICL. It seems that in ICL patient both inhibitory and apoptotic pathways are at play and coordinate with each other to suppress CD4 T cell proliferation leading to a lymphopenic condition.

## Figures and Tables

**Table 1:** T cell phenotype in idiopathic CD4 T cell lymphocytopenia (ICL) and 5 healthy controls (HC). Percentage of markers for activation (HLA-DR+), costimulatory and inhibitory molecules (CD28, CD80, CD86, CD152, PD-1, PDL-1), regulatory T cells (CD25+Foxp3+), apoptosis (AnnexinV+Propidium iodide, Fas, FasL), naïve T cells (CD54RA+CD62L+), and proliferation (Ki67+) expressed on CD4 and CD8 T cells from ICL patient and 5 healthy controls (HC 1-5). Percentage of variation (Var) between ICL and HC was calculated as follows: % Var = (ICL value − mean HC value)/mean HC value × 100.

Marker	CD4								CD8							
	**HC 1**	**HC 2**	**HC 3**	**HC 4**	**HC 5**	**Mean HC**	**ICL**	**% Var**	**HC 1**	**HC 2**	**HC 3**	**HC 4**	**HC 5**	**Mean HC**	**ICL**	**% Var**
**HLA-DR**	5.21	2.72	1.10	1.40	4.13	2.91	20.60	607.42	3.18	6.26	3.46	7.76	7.34	5.60	22.80	307.14
**CD152**	1.25	0.57	0.55	0.11	1.87	0.87	5.40	520.69	0.93	0.20	0.65	0.86	0.86	0.70	2.50	257.14
**PD-1**	8.39	10.80	16.20	19.00	6.12	12.10	64.00	428.84	8.19	13.10	22.10	16.60	13.70	14.74	47.60	222.97
**PDL-1**	0.80	2.20	0.60	1.79	0.61	1.20	5.80	383.33	1.87	2.20	2.90	0.84	2.04	1.97	4.02	104.06
**CD86**	1.07	0.91	0.53	0.72	1.10	0.87	3.90	350.35	0.61	0.48	0.45	0.46	3.50	1.10	4.60	318.18
**T-reg**	6.57	6.64	6.51	6.63	2.85	5.84	24.30	316.10								
**Annexin-V**	1.16	2.85	0.24	3.63	0.51	1.68	6.90	311.20	3.42	2.50	0.10	2.93	1.54	2.10	7.00	233.65
**FasL**	16.50	9.31	8.52	6.12	8.33	9.76	30.90	216.73	13.40	5.53	7.44	3.33	8.72	7.68	12.60	63.98
**Fas**	38.40	34.70	48.00	34.30	41.60	39.40	76.60	94.42	22.50	10.70	24.00	28.20	41.10	25.30	57.70	128.06
**CD80**	1.21	1.41	0.28	0.54	0.94	0.88	0.60	−31.51	3.22	0.19	1.02	1.46	1.19	1.42	4.00	182.49
**CD28**	97.50	97.30	97.90	95.10	92.80	96.12	45.30	−52.87	58.90	73.80	69.40	70.10	67.40	67.92	22.50	−66.87
**Naïve**	63.50	63.80	77.80	63.20	38.50	61.36	20.40	−66.75	67.00	44.20	53.80	47.00	39.30	50.26	52.90	5.25
**Ki67**	2.92	7.50	5.80	3.99	9.95	6.03	1.60	−73.47	1.01	9.59	5.34	0.56	5.03	4.31	1.60	−62.84

## Data Availability

The data presented in this study are available on request from the corresponding author. The data are not publicly available due to some unpublished content in the same file.
